# Transitions between epithelial and mesenchymal states during cell fate conversions

**DOI:** 10.1007/s13238-014-0064-x

**Published:** 2014-05-09

**Authors:** Xiang Li, Duanqing Pei, Hui Zheng

**Affiliations:** CAS Key Laboratory of Regenerative Biology, Guangdong Provincial Key Laboratory of Stem Cell and Regenerative Medicine, Guangzhou Institutes of Biomedicine and Health, Chinese Academy of Sciences, Guangzhou, 510530 China

**Keywords:** EMT, MET, cell states, cell fate conversion, iPSC generation, trans-differentiation, differentiation

## Abstract

Cell fate conversion is considered as the changing of one type of cells to another type including somatic cell reprogramming (de-differentiation), differentiation, and trans-differentiation. Epithelial and mesenchymal cells are two major types of cells and the transitions between these two cell states as epithelial-mesenchymal transition (EMT) and mesenchymal-epithelial transition (MET) have been observed during multiple cell fate conversions including embryonic development, tumor progression and somatic cell reprogramming. In addition, MET and sequential EMT-MET during the generation of induced pluripotent stem cells (iPSC) from fibroblasts have been reported recently. Such observation is consistent with multiple rounds of sequential EMT-MET during embryonic development which could be considered as a reversed process of reprogramming at least partially. Therefore in current review, we briefly discussed the potential roles played by EMT, MET, or even sequential EMT-MET during different kinds of cell fate conversions. We also provided some preliminary hypotheses on the mechanisms that connect cell state transitions and cell fate conversions based on results collected from cell cycle, epigenetic regulation, and stemness acquisition.

## INTRODUCTION

As two major types of cells in most animals, epithelial cells are known for their basement membrane, apical-basal axis of polarity, gap junction, immobility, and so on, while the characteristics of mesenchymal cells are almost just opposite, loosely associated, no polarity, and high mobility. Although the two types of cells are so different from each other, the transitions between epithelial and mesenchymal states, epithelial-mesenchymal transition (EMT) and mesenchymal-epithelial transition (MET), have been observed clearly and studied extensively during a variety of biological processes including embryonic development, cancer progression, and somatic cell reprogramming.

The first observation of EMT can be dated back to as early as 1890 when some ductal epithelial cells were described to acquire mesenchymal characteristics in breast tumors progression as reviewed previously (Nieto, 2011). It is suggested that EMT enables the tumor cells to acquire mesenchymal characteristics like loose cell-cell interaction and migratory ability, which further lead to the detachment of cells from tumor mass and the invasion of cells to other tissues. Although the requirement and significance of EMT for cancer progression has been debated for a long time, the migratory and invasive properties induced by EMT are widely accepted to be critical for metastasis (Peinado et al., 2007; Polyak and Weinberg, 2009).

EMT has been observed in multiple biological processes, especially embryonic development. The generation of adult tissues and organs requires multiple rounds of sequential EMT-MET, which is used to refer an EMT followed with its reversed process, MET (Thiery et al., 2009). For example, the formation and migration of mesenchymal cells is essential for the transformation from single-layered blastula into three-layered gastrula in various metazoans (Nakaya and Sheng, 2008). In addition, the development of heart has been considered as a good example for sequential EMT-MET. The formation of cardiac mesodermal cells during gastrulation and the organization of them into a two-layered epithelium later are considered as the first round of sequential EMT-MET. Another two rounds of sequential EMT-MET are observed during the folding around primitive foregut and the formation of four heart compartments (Nakajima et al., 2000; Thiery et al., 2009).

Since embryonic development can be considered as a combination of multiple differentiation processes from pluripotent or multipotent stem cells to somatic cells, and cancer progression is an acquisition of stem cells properties at least partially, it is reasonable to suggest the critical roles of EMT/MET or sequential EMT-MET during other kinds of cell fate conversions, which was summarized and discussed in the following sections of current review.

## EMT/MET DURING DIFFERENT CELL FATE CONVERSIONS

In general, the cell fate conversions are considered as the changes of cells from one type to another, and can be frequently observed during embryonic development when most types of cells are generated. The success of somatic cells to regain pluripotency after nuclear transfer or exogenous expression of four pluripotency-related transcriptional factors makes de-differentiation or iPSC generation as one new type of cell fate conversion (Gurdon, 1962a, b; Takahashi et al., 2007; Takahashi and Yamanaka, 2006). The direct reprogramming from fibroblasts to functional neurons enables cell fate conversion to cover an even wider area including differentiation, de-differentiation, and trans-differentiation (Vierbuchen et al., 2010). Since the combination of de-differentiation and differentiation or trans-differentiation alone can use cells isolated from patient skin or urine to generate patient-specific functional cells (Muller et al., 2009; Wang et al., 2012; Zhou et al., 2012), which have the potential to treat a variety of diseases without facing ethics concerns and immunologic rejections (Grabel, 2012; Nishikawa et al., 2008), a lot of efforts have been put to study the mechanisms underlying cell fate conversions to optimize these technologies for clinic application.

### EMT/MET during iPSC generation

In 2006, Dr. Yamanaka’s laboratory successfully induced mouse embryonic fibroblasts (MEF) into embryonic stem cells (ESC)-like cells with Oct4, Klf4, c-Myc, and Sox2. The generated cells were named induced pluripotent stem cells (iPSC). iPSC are able to generate tumors containing a variety of tissues from all three germ layers cells after being transplanted into nude mice and viable, fertile live-born progeny by tetraploid complementation (Takahashi and Yamanaka, 2006; Zhao et al., 2009). Because of the potential application of iPSC in clinic, the method to generate iPSC has been improved a lot since 2006 by using somatic cells other than fibroblasts for reprogramming (Aasen et al., 2008; Kim et al., 2008), by generating iPSC in other species like rat and pig (Esteban et al., 2009; Liao et al., 2009; Liu et al., 2008), by identifying new combinations of transcriptional factors (Maekawa et al., 2011; Yu et al., 2007), by developing new strategy to deliver transcriptional factors (Hou et al., 2013; Warren et al., 2010; Yu et al., 2009; Zhou et al., 2009), and by optimizing the culture systems (Chen et al., 2011; Chen et al., 2010a; Esteban et al., 2010).

During the generation of iPSC, multiple changes have been observed with the MEF, including those in gene expression profiles (Brambrink et al., 2008), epigenetic state (Koche et al., 2011; Watanabe et al., 2013), cell morphology (Li et al., 2010; Samavarchi-Tehrani et al., 2010), and cellular metabolism (Folmes et al., 2011). Among these changes, the transition of MEF from mesenchymal state to epithelial state has been recognized as a required step during the early phase of reprogramming (Li et al., 2010; Samavarchi-Tehrani et al., 2010). Inhibiting early MET by inducing EMT with transformation growth factor (TGF)-β or Snail1 prevents iPSC generation. In addition, Sox2 and c-Myc inhibit the expression of Snail1, TGF-β1, and TGF-β receptor 2 to suppress the mesenchymal characteristics of MEF, while Klf4 induces the epithelial properties by up-regulating E-cadherin directly (Li et al., 2010). Promoted MET has been used to explain the beneficial roles played by miR-302, Glis2, and several small molecules during iPSC generation (Chen et al., 2010b; Ichida et al., 2009; Maekawa et al., 2011; Subramanyam et al., 2011). Furthermore, the interactions between Vitamin C which increases the efficiency and quality for iPSC generation (Chen et al., 2012; Esteban and Pei, 2012; Esteban et al., 2010) and Ten-eleven translocation methylcytosine dioxygenase 1 (Tet1) which can replace Oct4 during iPSC generation (Gao et al., 2013) are also related to MET (Chen et al., 2013).

Although MET is necessary for fibroblasts reprogramming, there are still several questions remaining unclear. Firstly, since the process from MEF to iPSC can be considered as a reversed process of embryonic development at least partially, the reversed process of sequential EMT-MET, which is also a sequential EMT-MET, might be observed during iPSC generation. Secondly, the different or even opposite regulatory roles of the four transcriptional factors, like on TGFβ1 and TGFβ receptor 2, make MET induction more complex (Li et al., 2010). These two questions were answered in one of our recent publications (Liu et al., 2013a). Considering the complex functions of the four factors in regulating pluripotency (Kashyap et al., 2009; Pan et al., 2002) and the proper expression level of Oct4 required to maintain pluripotency (Pan et al., 2006), we proposed that the four factors have different functions during iPSC generation and may even have counteractions under certain circumstances or at certain time points. In order to diminish or decrease the possible counteractions, we introduced the four factors into MEF at different time points during iPSC generation.

**Figure 1:**
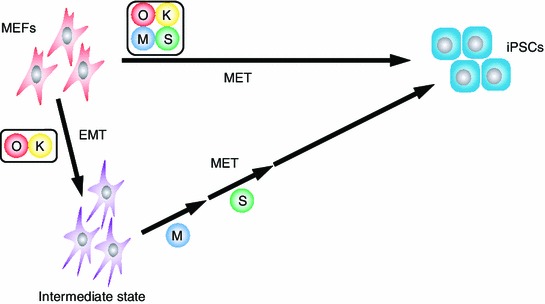
**Schematic illustration of EMT/MET during iPSC generation**. Simultaneous and time-dependent introduction of the four Yamanaka factor, Oct4 (O), Klf4 (K), c-Myc (M), and Sox2 (S) into MEF resulted in MET and sequential EMT-MET during the processes

Briefly, we determined reprogramming efficiencies with totally 74 different infection sequences. One particular infection sequence, Oct4 and Klf4 first, c-Myc next and Sox2 last, generated iPSC with the highest efficiency, about 600% of basal level (Liu et al., 2013a) (Fig. 1). After analyzing the differences between simultaneously infection (OKMS method) and the time-dependent infection of the four factors (OK+M+S method), we identified a temporary EMT in the early phase of iPSC generation as demonstrated by an impaired up-regulation of E-cadherin and temporary up-regulation of Snail2. Such temporary EMT was further confirmed with qPCR, Western blotting, FACS analysis, and wound healing assay (Liu et al., 2013a). Consistent with previous reports (Chiou et al., 2010; Liu et al., 2012), the short EMT and delayed MET were suggested to be resulted from the different abilities of Oct4 and Sox2/Klf4 to regulate the expression of E-cadherin and Snail2 (Liu et al., 2013a). In OKMS method, since the four factors were introduced simultaneously, the influences from Sox2 and Klf4 on Snail2 and E-cadherin were be larger than those from Oct4, which led to the consistent down-regulation of Snail2 and rapid up-regulation of E-cadherin. In OK+M+S method, since Sox2 was infected at least, Oct4 would overcome the counteractions from Klf4, which led to temporary up-regulation of Snail2 and impaired up-regulation of E-cadherin.

The reports on MET during iPSC generation in 2010 suggest this temporary EMT should generate more difficulties for later MET and decrease the reprogramming efficiency at the first glance (Li et al., 2010; Samavarchi-Tehrani et al., 2010). However, inducing EMT before MET with short treatment of TGFβ during OKMS reprogramming promoted reprogramming, while blocking EMT before MET with Repsox short treatment during OK+M+S reprogramming impaired reprogramming. Thus introducing a short EMT before MET is beneficial for iPSC generation, especially from MEF.

**Figure 2:**
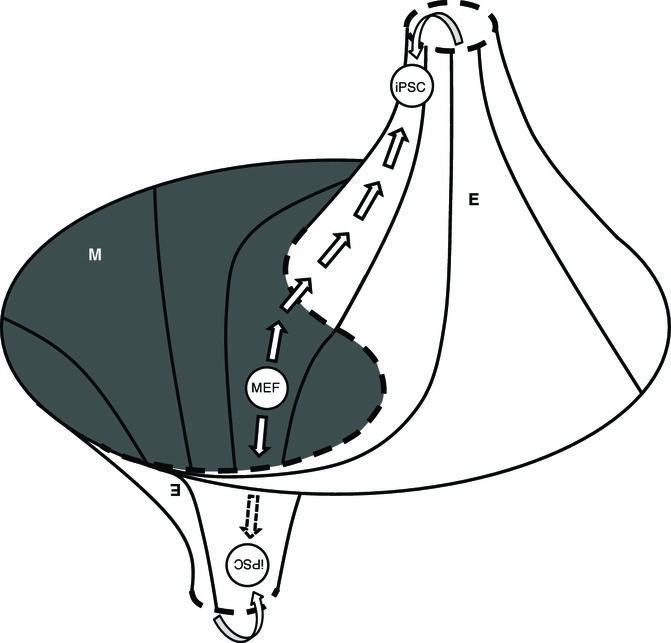
**Schematic illustration of the potential shortcut between optimal mesenchymal and epithelial states**. Mesenchymal state (M) and epithelial state (E) are collective concepts of two groups of cell states. The optimal mesenchymal state and optimal epithelial state are located in the middle of M and E, respectively. The shortcut was illustrated by putting the two optimal states on the reversed side of each other. MEF can be induced into iPSC by crossing the barriers between the two states. MEF can also be induced into the optimal mesenchymal state first and then to iPSC via optimal epithelial state

To explain how short EMT was induced on MEF which are already in mesenchymal state and why sequential EMT-MET promotes iPSC generation, we proposed a new model in previous report (Liu et al., 2013a). As two major cell states, both mesenchymal state and epithelial state are collective concepts and each covers a variety of distinct but similar cell states. We proposed that both of the two states have an optimal or central state within them, which can be named as optimal mesenchymal state and optimal epithelial state. The first hypothesis is to suppose a shortcut pathway between the optimal mesenchymal and epithelial states. When reprogrammed with OK+M+S method, MEF, though in mesenchymal state, would be converted into the optimal mesenchymal state after a short EMT, and took the shortcut pathway to the optimal epithelial state via a delayed MET and to iPSC finally (Fig. 2). When reprogrammed with OKMS method, MEF would not be able to take the advantages of the shortcut pathway and went a longer distance to epithelial state or iPSC, which makes its efficiency lower than that observed with OK+M+S method. This hypothesis was supported by the experiment to identify the optimal cell state for OKMS and OK+M+S reprogramming. When we used TGFβ/RepSox to induce EMT/MET in MEF and determined the ratio of the reprogramming efficiency of OK+M+S method to that of OKMS, we found that the highest ratio is in MEF with 12-h TGFβ treatment, and both longer treatment with TGFβ or Repsox treatment decreased the ratio.

Another available hypothesis is that the optimal mesenchymal state may be more suitable for the cell fate conversions, which is partially supported by the connection between EMT and stem cell characteristics (Hayashida et al., 2011; Mani et al., 2008). OK+M+S method-induced short EMT would convert MEF into this optimal mesenchymal state for easier reprogramming, whereas OKMS method could not fulfill this function. Of course, there should be other explanations for the ability of short EMT to promote MEF reprogramming, and how optimal mesenchymal and epithelial state are defined or inter-converted should be investigated in depth even if our hypotheses are right.

### EMT/MET during *in vitro* differentiation

Multiple rounds of sequential EMT-MET make embryonic development an excellent model and hot topic for EMT/MET research (Nieto, 2011; Thiery et al., 2009). However, the *in vitro* differentiation of ESC or iPSC is also useful for EMT/MET research because of their similarity to embryonic development and relative simplicity of the system. As a membrane marker for epithelial cells, E-cadherin has also been used as one of the markers for undifferentiated ESC (Li et al., 2012). Loss of E-cadherin expression, which suggests an EMT, can be observed immediately after ESC differentiation (Eastham et al., 2007). If considering EMT as an early step for ESC differentiation, MET should also be observed somewhere during the differentiation of ESC to epithelial cells.

Take the differentiation from iPSC to NSC as an example, immediate up-regulation of N-cadherin, a marker for mesenchymal cells, is essential for the efficient differentiation. However, E-cadherin expression is required to support the self-renewal of NSC (Karpowicz et al., 2009). Thus the expression switches between E-cadherin and N-cadherin, which suggests the transitions between epithelial and mesenchymal states (Gravdal et al., 2007; Maeda et al., 2005), might be observed multiple times during the differentiation from iPSC to NSC. In addition, MET has also been observed during the differentiation of hepatic stem/progenitor cells, suggesting the possibility to observe sequential EMT-MET during the differentiation from ESC/iPSC to hepatic cells (Li et al., 2011).

### EMT/MET during trans-differentiation

The successful trans-differentiation of somatic cells into functional neurons (Sheng et al., 2012a; Vierbuchen et al., 2010), NSC (Kim et al., 2011a; Sheng et al., 2012b; Wang et al., 2012), multilineage blood progenitors (Szabo et al., 2010), hepatocyte-like cells (Huang et al., 2011) or cardiomyocytes (Efe et al., 2011; Ieda et al., 2010) suggests a new route to generate target cells for transplantation without employing pluripotent stem cells as an intermediate state. The observation of EMT or MET during these trans-differentiation processes is greatly anticipated, not only because both the mesenchymal cells (fibroblasts) and epithelial cells (cells isolated from urine) have been used for trans-differentiation, but also because of the different characteristics received by the cells after trans-differentiation (Huang et al., 2011; Vierbuchen et al., 2010; Wang et al., 2012). Actually, if the cells were in different cell states (mesenchymal or epithelial) before and after cell fate conversions, EMT or MET should be observed during the conversions. Although the existence of sequential EMT-MET has not been reported yet, complex transitions between mesenchymal and epithelial state should exist during the NSC trans-differentiation for the similar reasons mentioned above and the critical functions of N-cadherin in neuron-neuron interaction (Tan et al., 2010).

## THE CONTRIBUTIONS OF EMT/MET TO CELL FATE CONVERSIONS

The observations of EMT/MET during different kinds of cell fate conversions do not enable us to answer the question that EMT/EMT is a by-product or a significant cause for cell fate conversions. Take MET during iPSC generation from MEF for example, MEF and iPSC definitely have the characteristics of mesenchymal and epithelial cells respectively. Thus the successful conversion from MEF to iPSC must be accompanied by a MET process. MET is demonstrated to be required for MEF reprogramming, because reprogramming was greatly impaired when EMT was induced or MET was inhibited (Li et al., 2010). However, this necessity might be explained by that cells will not become iPSC without epithelial characteristics.

One way to answer the question above is to study the reprogramming of epithelial cells. Ciliary body epithelial cells have been reported to have higher reprogramming efficiency to iPSC than fibroblasts (Ni et al., 2013). NSC, which require E-cadherin for self-renewal (Karpowicz et al., 2009), can be reprogrammed into iPSC with only two factors, Oct4 with Klf4 or c-Myc (Kim et al., 2008). In addition, cells isolated from urine have epithelial characteristics and have been considered as one excellent somatic cell types to generate iPSC or NSC not only because of their easy accessibility but also because of the high reprogramming efficiency (Wang et al., 2012; Zhou et al., 2011). These reports suggested that the MET during MEF reprogramming might only be a by-product rather than a significant cause. However, it is difficult to compare the reprogramming efficiencies between different types of somatic cells, because they have their own characteristics and can not be put at the same start point during reprogramming. Thus in the following text, we will discuss the connection between EMT/MET and other biological processes and how the marker events or factors during EMT/MET may contribute to cell fate conversions.

### Marker factors and events during EMT/MET

Unfortunately, the studies on MET are fewer than those on EMT not only because MET is regarded as a reversed and following process of EMT during embryonic development but also because both MET and EMT employ same regulatory system and most method to inhibit EMT can induce MET (Li et al., 2010; Liu et al., 2013a). The marker events during EMT, especially EMT during cancer progression, can be considered as the loss of tight cell-cell junction and the gain of migratory ability (Thiery, 2002). Thus the switch from E-cadherin to N-cadherin demonstrated with qPCR, immunoblotting or FACS analysis has been used to evaluate EMT (Gravdal et al., 2007; Liu et al., 2013a; Maeda et al., 2005), because E-cadherin plays critical role in maintaining the adhesion of epithelial cells (van Roy and Berx, 2008). Other membrane markers are also used to characterize epithelial and mesenchymal states, like epithelial cell adhesion molecule, epithelial keratins, Occludin, and Claudin3 for epithelial cells (Li et al., 2010; Litvinov et al., 1997), or Fibronectin and Vimentin for mesenchymal cells (Freire-de-Lima et al., 2011; Zhou et al., 2008). Many transcriptional factors, Snail1/2, Zeb1/2, Twist1/2, and E47, are also involved in EMT as reviewed extensively in previous reports (Nieto, 2011; Thiery et al., 2009). In addition, EMT is twisted with multiple signaling pathways. For example, TGFβ treatment can trigger both cell death and EMT (Massague, 2008), while modulating Snail1 expression can shift the balance between cell death and EMT (Franco et al., 2010). TGFβ molecules and receptors have also been used as markers for EMT (Li et al., 2010). EMT is also regulated by WNT/β-catenin (Li et al., 2013; Wu et al., 2012), FGF (Zhang et al., 2006), Sonic Hedgehog (Panman et al., 2006), mTOR (Lamouille et al., 2012), and other signaling pathways (Wang et al., 2010).

### EMT/MET and cell cycle

Cell cycle regulation is an old topic in cell biology and is observed during almost all kinds of cell fate conversions. iPSC have a high proliferation rate and short G_1_ phase similar to ESC (Neganova and Lako, 2008; White and Dalton, 2005). MEF gain these cell cycle signatures at the early phase of reprogramming. Proliferation induction and cell cycle arrest respectively promotes and impairs MEF reprogramming (Ruiz et al., 2010). In addition, these is a positive correlation between proliferation rate and reprogramming efficiency, which further confirmed the connection between cell cycle regulation and iPSC generation (Hanna et al., 2009).

On the other hand, EMT and cell cycle regulation are also inter-connected. TGFβ can lead to proliferation arrest and has extensive interaction with p53 (Adorno et al., 2009; Massague, 2008). Forced expression of p53 increased the mRNA levels of TGFβ1/2 (Fujiwara et al., 1994; Kannan et al., 2000). p53 is also essential for the responses to TGFβ signals by promoting the activation of multiple TGFβ target proteins (Cordenonsi et al., 2003; Termen et al., 2013). EMT induced by TGFβ in AML-12 cells is in a cell cycle-dependent manner and takes place at G_1_/S phase, where lies the major difference on cell cycle signatures between MEF and iPSC (Yang et al., 2006). In addition, another EMT inducer, Zeb1, also suppresses proliferation (Hugo et al., 2013), while p53 can suppress the expression of Zeb1 directly or via microRAN34/200c (Chang et al., 2011; Kim et al., 2011b). Therefore the decrease in reprogramming efficiency when TGFβ was included in the medium for iPSC generation might be mediated by the impaired proliferation (Li et al., 2010).

To explain the positive correlation between proliferation rate and reprogramming efficiency, Dr. Jaenisch’s laboratory has provided a model which suggested the early phase of MEF reprogramming was a stochastic process (Buganim et al., 2012; Hanna et al., 2009). That is each round of cell cycle would enable MEF to choose to enter iPSC cell fate or to stay in MEF cell fate. In addition, the inability of proliferation induction to increase reprogramming efficiency in y-axis when the number of cell cycle rather than the length of reprogramming time was used in x-axis suggests that the increase in the number of cell cycle accounts for almost all the effects of high proliferation rate on reprogramming (Hanna et al., 2009). This hypothesis is strongly supported by the short of observations with iPSC generated from cells in G_0_ phase.

The following question is what kind of events or markers during cell cycle promote reprogramming. One possibility is the DNA methylation regulation during cell cycle progression (Bou Kheir and Lund, 2010). There are multiple tasks like growth, expansion, and communication for cells in G_1_ phase, whereas cells are focused to DNA replication in S phase. Thus genes that specify the original cell fate would be silenced in S phase, as partially confirmed by the relative higher DNA methylation in S phase than G_1_ phase (Brown et al., 2007). The protein coded by these genes would be gradually degraded from S phase until the end of M phase, which resulted in a good opportunity for genes that specify different cell fates to function at the beginning of G_1_ phase. Since genes specify original cell fate is in a hypo-methylated state in contrast to the hyper-methylated state of genes specify other cell fates, the cells would keep their original fate without outside stimulants. However, when the expression of genes specifying for other cell fates were increased significantly like during iPSC generation and trans-differentiation, the possibility for cells to switch cell fate will increase significantly. In addition, it is also reasonable to propose that decreasing the overall DNA methylation might facilitate cell fate conversion by reducing the methylation levels on the genes specifying for other cell fates. This hypothesis is supported by the study on Tet1 and lysine-specific demethylase 1 (Chen et al., 2013; Gao et al., 2013; Lin et al., 2011). Over-expressing Tet1 increases the reprogramming efficiency, possibly by reducing DNA methylation.

### EMT/MET and histone modifications

As just discussed on DNA methylation, histone modifications are also required to be duplicated and inherited during cell cycle progression (Probst et al., 2009). Thus histone modifications may also be possible to mediate the interaction between cell cycle and cell fate conversions. However, the complex regulation on histone modifications during cell cycle and undetermined functions of different histone modifications make our discussion more difficult.

The histone modifications affect cell fate conversions from a variety of aspects (Papp and Plath, 2012). Increasing H3K4 methylation with lysine-specific demethylase 1 inhibitor, decreasing H3K9 methylation with Setdb1, reducing H3K27 methylation with Utx, and inhibiting H3K36 methylation with Jhdm1a/1b are all reported to benefit iPSC generation (Chen et al., 2012; Mansour et al., 2012; Wang et al., 2011a; Wang et al., 2011b). Thus, by affecting cell cycle, EMT/MET may influence histone modifications and subsequent cell fate conversion. Although difficult to eliminate the participation of cell cycle, there are many reports on the interactions between EMT/MET and histone modifications. Histone deacetylases 3 not only increases H3K4 methylation by interacting with hypoxia-induced WD repeat-containing protein 5 to activate mesenchymal gene expression, but also serves as an essential co-repressor to repress epithelial gene expression (Wu et al., 2011). LSD1 which functions in the demethylation of H3K4 and H3K9 not only serves in NuRD complex to block EMT during breast cancer metastasis (Wang et al., 2009), but also forms complex with Snail1 and Twist to repress E-cadherin expression (Fu et al., 2011; Lin et al., 2010). Considering the extensive interactions between EMT/MET and histone modifications as reviewed (Nieto, 2011), it is reasonable to hypothesize that EMT/MET may contribute to cell fate conversions by modulating histone modifications and other epigenetic properties.

### EMT/MET and stemness

As mentioned above, EMT was first observed in breast cancer and plays critical roles during cancer progression (Thiery et al., 2009). On the other hand, cancer stem cells (CSCs) are characterized as a special group of cancer cells with enhanced tumorigenicity, partial stemness as in self-renewal and differentiation, and CD44^+^/CD24^−/low^ on cell surface (Al-Hajj et al., 2003; Dalerba et al., 2007). These two concepts, EMT and CSCs, are not well connected until the report by Mani et al. in 2008. The induced EMT in human mammary epithelial cells increased the CD44^+^/CD24^−/low^ population with properties associated with mammary epithelial stem cells like abilities to form mammosphere and to differentiate into myoepithelial or luminal epithelial cells (Mani et al., 2008). The connection between EMT and CSCs is further established with the common characteristics between cells undergoing EMT and CSCs in TGFβ signaling pathway activation, circulation in blood, and chemo-resistance as reviewed (Hayashida et al., 2011). Thus it seems that EMT may be able to induce stemness in cells at least during cancer progression.

However, the studies on ESC and iPSC are not fully consistent with the hypothesis above. ESC as isolated from inner cell mass are considered to be typical epithelial cells. The epithelial marker, E-cadherin, together with proteins like SSEA1, alkaline phosphatase, Oct4, Nanog, and others have been used to determine the undifferentiated state of ESC (Horie et al., 2010; Redmer et al., 2011). The expression of E-cadherin in undifferentiated ESC decreased immediately after the induction of differentiation (D’Amour et al., 2005; Eastham et al., 2007). In addition, blocking the up-regulation of E-cadherin significantly decreased the efficiency during iPSC generation from MEF (Li et al., 2010). Although the absolute requirement of E-cadherin for pluripotency is still under debate (Eastham et al., 2007; Ullmann et al., 2007), E-cadherin contributes to the survival, self renewal, and pluripotency of ESC at least partially (Li et al., 2012). Thus it seems that EMT may prohibit stemness in cells like iPSC and ESC. The opposite influences of EMT on stemness observed in the two sets of studies above suggest that either EMT has multiple roles or different aspects of EMT function differentially in regulating stemness.

## FUTURE DIRECTIONS

We have provided a brief review on the connection between EMT/MET and cell cycle, epigenetic regulation, and stemness to provide possible mechanisms underlying the contributions of EMT/MET to cell fate conversions. Of course, EMT/MET also twists with other biological processes or factors including cell senescence with telomerase reverse transcriptase (Qiao et al., 2012; Yu et al., 2014), hypoxia with hypoxia induced factor 1α (Liu et al., 2013b; Marie-Egyptienne et al., 2013), microRNAs like miR-200 (Ocana and Nieto, 2008; Park et al., 2008), and so on, we did not listed them all in current review. Since the interactions among these biological processes are also complex as those between cell cycle and epigenetic regulation mentioned above, the future study on how EMT/MET contributes to cell fate conversions should not be limited in one or few biological processes.

In our opinion, the well established connections between EMT/MET and large amount of biological processes is the major problem faced by researchers who intend to study the contributions of EMT/MET to cell fate conversions. Firstly, the complex interactions among different biological processes make it difficult to study one aspect at one time. For example, cell cycle, DNA methylation, and tumor progresses have been suggested to function together under certain circumstances (Robertson et al., 2000; Wu et al., 1993). Secondly, these complex interactions lead to opposite observations on how EMT/MET affect cell fate conversions under different conditions. TGFβ treatment in whole process of iPSC generation from MEF decreased reprogramming efficiency, while pre-treatment or short treatment (Day 0–1.5 during reprogramming) with TGFβ decreased the proliferation rate and significantly increased the reprogramming efficiency. (Li et al., 2010; Liu et al., 2013a). The studies on EMT during CSCs and iPSC generation suggested the opposite roles of EMT on stemness as mentioned above. The ability of periphery cells around undifferentiated ESC to have both mesenchymal characteristics and ESC properties makes the correlation between EMT and stemness even more complex (Ullmann et al., 2007). Thirdly but not lastly, different biological processes have distinct preference with different aspects of EMT/MET. For example, the function of E-cadherin during EMT/MET may have a closer connection with cell-cell adhesion and WNT/β-catenin pathway (Tian et al., 2011; van Roy and Berx, 2008), while TGFβ may prefer to interact with p53 (Dupont et al., 2004; McPherson, 1996).

To solve the problem above, one possibility is to put EMT and MET together and study sequential EMT-MET during cell fate conversions. Sequential EMT-MET has been observed during embryonic development and iPSC generation (Liu et al., 2013a; Thiery et al., 2009). In addition, EMT has long been considered important during the initial phase of metastasis, MET is also suggested to be important for the latter phase of metastasis, when cancer cells regain similarities to primary tumors at the secondary sites (Chaffer et al., 2007). Although sequential EMT-MET has not been reported during differentiation and trans-differentiation, it is reasonable to suggest that inducing sequential EMT-MET in proper manner may facilitate different cell fate conversions.

To explain why temporary EMT before EMT promotes iPSC generation from MEF, we proposed a hypothesized model with optimal mesenchymal and optimal epithelial states as above. Considering the sequential EMT-MET, the preliminary hypothesis that an optimal mesenchymal may function as intermediate for efficient cell fate conversions might be more reasonable. This hypothesis is able to explain the beneficial effects of temporary EMT during iPSC generation and the opposite roles of EMT on stemness regulation. The hypothesis is supported by the chromosomal instability during EMT and in mesenchymal stem cells (Miura et al., 2006; Roschke et al., 2008) and challenged by the short of evidences during a variety of cell fate conversions.

## ABBREVIATIONS

EMT: epithelial-mesenchymal transition
ESC: embryonic stem cells
iPSC: induced pluripotent stem cells
MEF: mouse embryonic fibroblasts
MET: mesenchymal-epithelial transition
OKMS: Oct4, Klf4, c-Myc and Sox2 were introduced into MEF simultaneously during reprogramming; OK+M+S; Oct4 and Klf4 were first, c-Myc was next, and Sox2 was last introduced into MEF during reprogramming
Tet1: Ten-eleven translocation methylcytosine dioxygenase 1
